# Integrating clinical, molecular, proteomic and histopathological data within the tissue context: *tissunomics*

**DOI:** 10.1111/his.13828

**Published:** 2019-05-14

**Authors:** Santiago Ramón y Cajal, Stefan Hümmer, Vicente Peg, Xavier M Guiu, Inés De Torres, Josep Castellvi, Elena Martinez‐Saez, Javier Hernandez‐Losa

**Affiliations:** ^1^ Translational Molecular Pathology Vall d'Hebron Institute of Research (VHIR) Universitat Autònoma de Barcelona Barcelona Spain; ^2^ Department of Pathology Vall d'Hebron University Hospital Barcelona Spain; ^3^ Spanish Biomedical Research Network Centre in Oncology (CIBERONC) Barcelona Spain; ^4^ Department of Pathology Bellvitge University Hospital Barcelona Spain

**Keywords:** genomics, histopathology, proteomics, tumour heterogeneity

## Abstract

Malignant tumours show a marked degree of morphological, molecular and proteomic heterogeneity. This variability is closely related to microenvironmental factors and the location of the tumour. The activation of genetic alterations is very tissue‐dependent and only few tumours have distinct genetic alterations. Importantly, the activation state of proteins and signaling factors is heterogeneous in the primary tumour and in metastases and recurrences. The molecular diagnosis based only on genetic alterations can lead to treatments with unpredictable responses, depending on the tumour location, such as the tumour response in melanomas versus colon carcinomas with BRAF mutations. Therefore, we understand that the correct evaluation of tumours requires a system that integrates both morphological, molecular and protein information in a clinical and pathological context, where intratumoral heterogeneity can be assessed. Thus, we propose the term ‘tissunomics’, where the diagnosis will be contextualised in each tumour based on the complementation of the pathological, molecular, protein expression, environmental cells and clinical data.

## General Background of Tumour Pathology

Despite constant progress in cancer research, mortality rates remain high.^1^, ^2^ Five‐year survival is greater than 80% for carcinoma of the breast, prostate and bladder, cutaneous melanoma and testicular and thyroid tumours, but less than 10% in pancreatic, liver, oesophageal, lung and stomach tumours and glioblastoma. If we focus on tumour stage, we observe that in luminal breast cancer, expected survival is greater than 90% in patients without metastases at diagnosis and less than 25% in patients with disseminated disease.^3^ With this in mind, early detection, histological typing and extent of dissemination are critical for treatment success. Furthermore, the more than 250 tumour types and hundreds of subtypes, on a background of thousands of genetic, epigenetic and microRNA alterations,^4^, ^5^, ^6^ highlight the importance of precise tumour sample evaluation in deciding the most appropriate clinical treatment.

Inter‐ and intratumour heterogeneity (ITH)^7^, ^8^ act as a critical barrier to improving therapeutics: most of the targets and biomarkers approved by the United States Food and Drug Administration are not expressed uniformly throughout tumour tissue; for example, the human epidermal growth factor receptor 2 (HER2) in gastric adenocarcinoma,^9^, ^10^ progesterone and oestrogen receptors in breast tumours (considered positive when detected in 1% of tumour cells)^11^ and EML4–ALK translocation in lung adenocarcinoma (threshold of 15%),^12^ among others.^13^, ^14^

## Current Situation and Challenges

### Why Are Malignant Tumours Heterogeneous?

The paradigm of cancer as a genetic disease has existed since the early 1970s. For the last 50 years, cancer has been explained according to the somatic mutation theory (SMT),^15^ which states that genetic mutations accumulate in cells by inducing clonal expansion. Following Darwinian‐type evolution, an initial clone accumulates multiple mutations, resulting in malignant transformation characterised by growth independence and resistance to apoptosis. These clones have trunk and branch mutations, and may also have driver and passenger mutations.^16^, ^17^, ^18^ During tumour progression and after chemotherapy or radiotherapy, genetic alterations accumulate to select for the best‐adapted clone. Molecular heterogeneity has been described in renal adenocarcinoma, breast cancer and glioblastoma, among others.^19^ It is thought that these clones with greater proliferative advantages and resistance to local stresses then predominate.^20^, ^21^, ^22^, ^23^, ^24^

Other theories include the tissue organisation field theory (TOFT), which postulates that neoplasia arises from a local tissue disorder and may be due to a defect in interaction between cells and other tissue components,^15^, ^25^, ^26^, ^27^ where epigenetic or genetic changes affect both epithelial and stromal cells in multiple locations. Grouping together the evidence for these theories, it has been demonstrated that (i) many spontaneous somatic mutations accumulate in cells during their lifetime, (ii) oncogenes and tumour suppressor genes play a critical role in cancer development and (iii) environmental factors trigger cancer onset and progression.^15^ Regarding field cancerisation, some authors have proposed that cancer is a tissue disorder with similarities to abnormal embryonic development,^15^, ^25^ while others propose that cancer is a consortium of clones and local factors.^28^ It is understood that a single clone cannot harbour all the genetic alterations required for the invasive and metastatic tumour phenotype and that several clones acquire malignant properties that, synergistically, allow cells to grow, invade and metastasise. This consortium of tumour cell clones and microenvironment cells includes leucocytes, lymphocytes, fibroblasts and endothelial cells. A minimum number of cells is also required for a clone to be biologically viable – a phenomenon known as the Allee effect. This clonal cooperation has been proposed in pancreatic and breast cancer and melanoma and for circulating tumour cell (CTC) clusters.^29^, ^30^, ^31^, ^32^, ^33^

### Some Oncogenic Alterations Are Tissue‐Specific

Certain genetic alterations are only observed in the context of specific cell or tissue types;^34^ for example, alterations in the APC gene in colorectal cancer, CDH1 (E‐cadherin) in gastric carcinoma, the VHL gene in renal carcinoma or BCR/ABL in leukaemia.^35^, ^36^ These specific genetic alterations demonstrate that tissue type plays a role, which is currently not well understood and is under intensive investigation. The higher incidence of certain somatic mutations in some cell types might be explained by a cell‐ or tissue‐specific chromosome disposition during interphase. This, along with epigenetic imprinting, may serve as a mechanism of gene regulation and allow differential access of carcinogens to certain DNA regions, resulting in a higher probability of mutations and specific rearrangements in some cells but not others. Similarly, the accessibility of certain chromosomal regions might explain cell‐type‐specific chromosomal translocations such as BCR/ABL in leucocytes or VHL mutations in renal cell carcinoma.^37^, ^38^ Some mutations and genetic alterations are uniformly present in the entire organism, such as germline mutations, BRCA1 and BRCA2, VHL in Von Hippel Lindau syndrome and as many as 30 others, but they are usually only associated with tumours in specific tissues, such as BRCA1/2 in breast and ovarian tumours.^39^ This clearly indicates that driver, trigger or passenger mutations only exert an effect in certain tissues.

### Some Histopathological Features Suggest Oncogenic Alterations

The specific morphological patterns observed in human tumours have helped to identify distinct genetic changes: for example, characteristic chromosomal translocations have been identified in desmoplastic round‐cell tumours, clear‐cell sarcoma, synovial sarcoma and rhabdoid tumours.^40^, ^41^, ^42^ Moreover, a simple haematoxylin and eosin stain can indicate the presence of a number of hereditary tumours with specific genetic alterations, such as medullary thyroid carcinoma, multiple endocrine neoplasia (MEN),^43^, ^44^ the cribriform‐morular variant of papillary thyroid carcinoma,^45^ familial adenomatous polyposis,^46^ medullary breast carcinoma, familial breast cancer associated with specific BRCA1 mutations,^47^ renal carcinoma, associated with SDHB mutations which shows flocculent cytoplasmic vacuoles,^48^, ^49^ hereditary leiomyomatosis and renal cell cancer syndrome,^50^ and sebaceous lesions associated with Muir–Torre syndrome in colon cancer.^51^, ^52^ Thus, hereditary cancer predisposition syndromes can be identified by immunohistochemistry, which has emerged as a cost‐effective strategy (see Table 1).^53^

**Table 1:** Molecular alterations detected by immunohistochemistry (IHC) analysis in tumour samples

Molecular alterations in tumors	Principles of immunohistochemical staining in tumoral cells	Examples
Chromosomal translocation	Overexpression of protein encoded by one of the genes involved in fusion or chimeric protein encoded by the two genes involved in fusion. Positive immunostaining in tumour cells	BCL2 expression in follicular lymphoma
		Cyclin D1 expression in mantle cell lymphoma
		ALK expression in different tumour cells (anaplastic lymphoma, NSCLC)
		NUT expression in NUT midline carcinoma
		ERG expression in prostate carcinoma
		ROS1 expression in NSCLC
Gene mutation	Aberrant subcellular localisation of antigen or protein	Nuclear translocation of B‐catenin in (CRC, desmoid fibromatosis, cribriform morular variant of PTC)
		Cytoplasmic expression of nucleophosmin in AML
		Cytoplasmic expression of BRCA1 in BC.
	Stabilisation and strong expression of the protein encoded by the gene mutated	P53 in different tumour types (high grade serous ovary carcinoma)
	Mutation specific antibodies, the antibody do not recognize the WT form of the protein (gene)	IDH1 R132H expression in gliomas
		EGFR L858R expression in NSCLC
		EGFR Del 19 expression in NSCLC
		BRAF V600E expression in different tumor types (Melanoma, PTC, CRC, others.)
	Presence or lack of mutation of certain genes give an overexpression of certain surrogate markers	LCC without IGH mutation is associate with overexpression of ZAP‐70
Gene deletion or loss of function	Inactivating mutation, deletion or promoter hypermethylation of gene gives a loss of protein expression of the encoded genes	Loss of E‐cadherin staining in lobular breast carcinoma
		Loss of INI‐1 staining in rhabdoid tumor, and epithelioid sarcoma
		Loss of staining for any MMR proteins (MLH1, MSH2, MSH6, PMS2) in HNPCC with MSI
		Loss of staining for parafibromin in parathyroid Carcinoma
		Loss of staining for Rb in spindle cell /pleomorphic lipoma
		Lack of staining for SDHB in hereditary paragangliomas and gastrointestinal stromal tumor
		Loss of PTEN expression in endometrial cancer with PTEN mutation and Cowden Syndrome
Gene amplification	Increase in copy number of gene gives an overexpression of the encoded protein	HER2 strong staining in breast and gastric cancer associated with HER2 amplification
		Strong staining for MDM2 or CDK4 associates with 12q14‐15 amplification in liposarcoma or well‐differentiated osteosarcoma.
		Strong MET expression in NSCLC

Adapted from Chan *et al*.^53^^.^

In contrast, other molecular alterations have been associated with tumours whose morphological characteristics are strikingly distinct, such as (i) the ETV6–NTRK3 translocation, detected in diverse tumour types including infantile fibrosarcoma, cellular mesoblastic nephroma and secretory breast carcinoma;^54^ (ii) translocation affecting the ALK gene, in anaplastic large‐cell lymphoma, lung adenocarcinoma, inflammatory myofibroblastic tumour and others;^55^ (iii) EWSR1–CREB1 translocation in clear‐cell sarcoma and angiomatoid fibrous histiocytoma;^56^ and (iv) BRAF mutations and translocations in benign nevi, malignant melanoma, colon adenocarcinoma, glioblastomas and pilocytic astrocytoma, as well as others.^57^

Thus, the association between genetic alterations and specific tumour types is not clear, but some histopathological patterns suggest certain genetic alterations and hereditary tumour syndromes.

## Problems and Barriers

### Bias and Limitations of Molecular Classification and Somatic Theory

#### Intratumour heterogeneity and pitfalls in the interpretation of oncogenic factors in small biopsies

**Figure 1:**
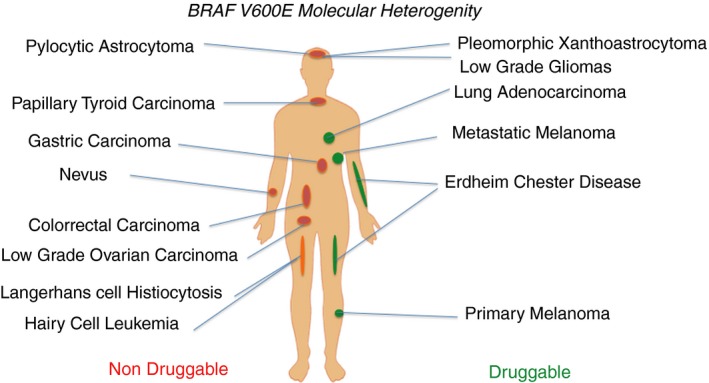
Genetic alterations such as BRAF mutations can be detected in many different tumours. Importantly, the biological meaning and the response to specific BRAF inhibitors depend on tumour type and tissue localisation.

Intratumour heterogeneity at the morphological, molecular and proteomic level is seen in most tumours. In melanoma, intratumour heterogeneity of BRAF mutations is described in more than 10% of cases^11^, ^13^, ^14^, ^58^ so, depending on the zone biopsied within a tumour, the determination of BRAF mutations may be wild‐type or mutant, with clear clinical implications (see Figure 1). This example can be extended to other targets and biomarkers, such as EGFR mutations in non‐small cell lung cancer (NSCLC),^59^ ALK rearrangement and others.^60^

#### Molecular and preneoplastic changes in the context of the adjacent tissue: field cancerisation

Until now, tumour diagnosis has focused on histological and molecular features, yet it is well known that the surrounding cells and other areas of the tissue may display premalignant histological changes such as dysplasia; furthermore, many genetic alterations occur without cytological changes. These environmental changes can affect tumour progression by exerting evolutionary pressure on tumour cells and ultimately determining which mutations are selected.^27^ Examples of preneoplastic histological lesions include dysplasia (cytological atypia, large hyperchromatic nuclei or nucleoli) and *in‐situ* carcinomas (with no basement membrane invasion), which are observed in most carcinomas. It is noteworthy that some early neoplastic lesions such as colon adenomas and skin naevi are polyclonal. Progression to cancer probably involves the accumulation of multiple field cancerisation driver mutations among synergistically acting groups of mutations.^27^ In this regard, molecular studies of peritumour areas in normal ducts of the breast or prostate may show a high number of genetic alterations and driver mutations.^61^ The clinical meaning of these molecular changes remains unknown, and further large studies are needed to establish their correlation with tumour relapse. Nonetheless, two important conclusions can be drawn: (i) the study of non‐tumour tissue can be relevant in predicting prognosis and risk of second or multiple neoplasia and (ii) we must be very cautious making a diagnosis of malignancy based only on molecular data, as not all mutations in, for example, driver genes, are directly linked to cancer development.

#### Mixed tumours as an example of the relevance of integrating molecular features into their morphological context

Mixed tumours provide the most extreme examples of intratumour heterogeneity. They are composed of two or more histological components, each with a different natural history, and are occasionally detected in every organ of the human body.

In most cases, the two biologically distinct tumour components are clonally related, and it is thought that the more aggressive component arises from the more indolent one and is usually responsible for the aggressiveness of the tumour. The amount of each component is also closely related to outcome.

In a small proportion of cases the two components are not clonally related: they are not true mixed tumours, but collision tumours in which two distinct neoplasms have developed independently, with a different natural history.

In a high proportion of mixed tumours, morphological appearance correlates with molecular diversity. For example, mixed endometrioid and serous carcinomas of the endometrium contain elements with the microscopic characteristics of endometrioid and serous carcinoma, and molecular analysis of microdissected tumour tissue shows that the two components have a different mutational profile. Other mixed tumours are ambiguous, and different microscopic features develop in tumour components with similar molecular alterations, probably as a result of interaction with the microenvironment.^62^

In some mixed tumours the microscopic diversity is extreme. This is the case in biphasic synovial sarcoma, carcinosarcomas and some mesotheliomas. In carcinosarcomas (named differently in different organs: metaplastic carcinoma, sarcomatoid carcinoma, carcinosarcoma) the malignant epithelial component gives rise to mesenchymal elements, and may even contain fully developed sarcomatoid features, such as malignant skeletal muscle, cartilage or bone elements. Integration of the molecular features into the appropriate microscopic context is essential to understand these tumours and accurately assess prognosis.

#### Cancer‐type‐independent molecular classification of therapeutic targets

Currently, precision oncology is based on the existence of alterations in certain specific therapeutic targets regardless of tumour location or type. Tumours with somatic mutations such as BRAF, EGFR or gene rearrangements such as ALK are considered therapeutic options regardless of tumour type or location, and tumours may even be characterised as BRAFomas or ALKomas. However, some clinical data have shown that, while BRAF inhibitors are effective in melanoma, the same inhibitors of the same BRAF mutations in colorectal cancer induce feedback loops in signalling pathways that can have a detrimental effect.^63^, ^64^ Cell signalling pathways are also highly regulated, often dependent on numerous positive and negative feedback loops, which are specific to the cell type and tumour environment. For this reason, BRAF inhibition in colorectal cancer indirectly results in EGFR receptor activation and subsequent PI3K–Akt pathway activation. Similarly, ALK alterations and their inhibitors do not have the same clinical response in lung tumours as in neuroblastomas.^65^ Further examples are inhibitors of EGFR, HER2 and HER3, depending on the tumour type and location. In a recent basket clinical trial, not all tumours were affected by the same HER mutations, and response to the HER2 inhibitor (neratinib) varied in different tissues: for example, colorectal or bladder cancer with HER2 mutations did not respond.^66^ In summary, specific drug treatment depends on the tissue context.^34^

#### Assessment and validation of molecular subtypes by protein expression

Molecular classification based on mRNA expression profiling of gene groups is a frequent approach to identify and classify molecular subtypes of tumours. One of the major achievements of molecular classification has been the subcategorisation according to prognosis, and it is instrumental for the identification of new genetic alterations. However, mRNA‐based expression studies are often limited by a low reproducibility, depending on mRNA stability. Furthermore, single‐cell sequencing approaches are not feasible in routine diagnostics. Currently, samples are sequenced as the bulk of tumour cells and therefore do not provide information on ITH. In addition to these technical issues, monitoring gene expression at the mRNA level does not necessarily correlate with the function of a protein within a cell, as many post‐transcriptional modifications determine whether or not mRNA finally gives rise to a functional protein. Importantly, however, the different molecular subtypes based on enormous mRNA expression matrices can be validated according to protein or gene expression using just a few *in‐situ* hybridisation or IHC probes in most molecular cancer subtypes (see Table 2).

**Table 2:** Molecular signatures detected by IHC and FISH analysis in FFPE tumour samples

Tumour type	Molecular signature Classification	Immunohistochemistry	FISH
Breast cancer	Luminal A	ER+, PR+, GATA+, FOXM+, Ki67 <15%	
	Luminal B	ER+, PR+, GATA+, FOXM+, Ki67 >15%	
	HER2 enriched	ER−, PR−, HER2+	ERB2 ampl.
	Basal‐like	ER−, PR−, HER2−, CK5+, EGFR+	
	Claudin low	ER−, PR−, HER2−, CD44+, Snail+	
	Normal breast‐like	ER−, PR−, HER2−, CD36+	
Colon cancer	CMS1 (immune)	FRMD6−, HTR2B−, ZEB1−, CDX2+	
	CMS2	FRMD6−, HTR2B−, ZEB1−, CDX2+	
	CMS3	FRMD6−, HTR2B−, ZEB1−, CDX2+	
	CMS4 (mesenchymal)	FRMD6+, HTR2B+, ZEB1+ CDX2−	
Glioblastomas	Proneural	IDH mut, p53mut, OLIG2	PDGFRA ampl, CDK4 ampl, CDK6 ampl
	Mesenchymal	CD44, NF1	EGFR ampl
	Classical	EGFR vIII mut, p53−	EGFR ampl, loss of PTEN and CDKN2A
	Neural	–	
Medulloblastoma	WNT‐activated	β‐Catenin nuclear, GAB1−, YAP1+, Filamin A+	Monosomy chr. 6
	SHH‐activated, TP53‐WT	β‐Catenin cyto, GAB1+, YAP1+, Filamin A+	GLKi1 ampl, PTCH1 del
	SHH‐activated, TP53‐mut	β‐Catenin cyto, GAB1+, YAP1+, Filamin A+, p53+	MYCN ampl, CLI2 ampl, 17p loss
	Group 3 (Gabaergic)	β‐Catenin cyto, GAB1−, YAP1−, Filamin A−	17q ampl
	Group 4 (Glutaminergic)	β‐Catenin cyto, GAB1−, YAP1−, Filamin A−	MYC ampl, CDK6 ampl
Endometrial	Ultramutated (POLE)		
	Hypermutated (MSI)	MMRd (MLH1−)	
	Copy number low		
	Copy number high	P53+	

IHC, Immunohistochemistry; FISH, Fluorescence *in‐situ* hybridisation; FFPE, Formalin‐fixed paraffin‐embedded.

##### Breast carcinoma

According to the mRNA expression profile, six molecular subtypes are described: luminal A, luminal B, HER2+, basal‐like, claudin‐low and normal breast‐like.^67^, ^68^ However, RNA signatures can vary dramatically between patients with the same molecular subtype^69^ and, with the exception of the basal subtype, are not highly reproducible for diagnosis on microarray analysis.^67^, ^70^, ^71^ A high degree of ITH has been described, as assessed by immunohistochemical (IHC) studies,^72^ array‐CGH studies^71^ and massive parallel sequencing technologies.^73^ Given these problems in the exact diagnosis of breast cancer subtype by molecular characterisation, IHC‐based characterisations are recommended as a diagnostic approach in most of the consensus oncology guidelines.^74^ Additional IHC staining helps to distinguish luminal A and B subtypes, such as GATA3 and FOXM.^75^ CK5 and EGFR expression define the basal subtype of breast cancer,^76^ and CD44 and Snail expression determine the claudin‐low subtype.^77^ Finally, the normal breast‐like subtype is defined by low expression of the above‐mentioned markers and expression of factors such as CD36 in a low‐grade histological tumour.^78^

##### Colorectal carcinoma

Molecular subtypes of colorectal carcinoma have been distinguished by genomic, epigenetic and transcriptomic parameters.^79^, ^80^ Four different colorectal carcinomas, CMS1–4, have been described according to immunohistochemistry. The molecular subtypes can be identified as mesenchymal type (CMS4), which has the worst prognosis, immune type (CMS1) and epithelial subtypes (CMS2/3), based on four proteins: CDX2, FRMD6, HTR2B and ZEB1. In all subtypes, the drivers and actionable genes must be studied according to approved protocols (e.g. RAS, BRAF).^81^, ^82^

##### Lung carcinoma

Three molecular subtypes have been described based on DNA methylation [high, intermediate or low CpG island methylator phenotype (CIMP)], transcriptomics and RNA array: proximal proliferative, proximal inflammatory and terminal respiratory unit.^83^ Recently, using an integrative analysis with all available omics data, six different molecular subtypes have been described.^84^ Protein markers to distinguish those subtypes are still under way and that classification is not used in clinical oncology guides.

Nevertheless, druggable genetic alterations can be observed in the different molecular subtypes, and current oncology guidelines follow a reasonable scheme based on druggable genes, combining polymerase chain reaction (PCR)‐based methods to identify EGFR and BRAF mutations, and *in‐situ* hybridisation or immunohistochemistry for the expression of BRAF V600E, ALK, ROS, HER2, RET and MET.

##### Glioblastomas

Four molecular subtypes have been described based on gene expression profile: proneural, mesenchymal, mixed and unknown,^85^ and up to six based on methylation arrays, recurrent driver genes and transcriptomics, by the DKFZ (Deutsches Krebsforschungs‐zentrum).^86^, ^87^, ^88^, ^89^ These molecular subtypes are used currently in clinical oncology guides. In brief, according to the most recent World Health Organisation (WHO) classification,^90^ grade IV gliomas are classified as IDH‐mutant GB, IDH‐wt GB and midline glioma with H3 K27M mutation. In order to discriminate between subtypes the following can be analysed using IHC, fluorescence *in‐situ* hybridisation (FISH) or PCR: IDH1/IDH2, ATRX, TP53, FGFR, NF1, H3.1 and H3.3 mutations, EGFR and PDGFR amplifications, BRAF V600 mutation or translocations and CDKN2A/CDKN2B deletion.

##### Medulloblastomas

Medulloblastomas have four molecular subtypes with clear clinical differences (group 1 WNT activated; group 2 SHH activated; group 3 or GABAergic; and group 4 or glutaminergic). These subtypes can be assessed by immunohistochemistry or FISH, studying β‐catenin, GAB1, YAP1, Filamin A, p53, Glki1, 17q+ (group 3), MYC and CDK6 amplification.^91^, ^92^, ^93^, ^94^

##### Endometrial carcinoma

Two types of endometrial carcinoma have been described based on a combination of epidemiological, clinical, histological and molecular genetic data, distinguishing type I endometrioid endometrial carcinoma (EEC) and type II non‐endometrioid endometrial carcinoma (NEEC).^95^ A molecular classification of EC has been proposed based on The Cancer Genome Atlas Research Network (TCGA) study, published in 2013. TCGA analysis revealed four tumour groups:^96^ group 1, EEC with somatic inactivating mutations in POLE exonuclease and very high mutation rates (hypermutated) (7%); group 2, EEC with microsatellite instability (MSI), frequently with MLH‐1 promoter hypermethylation and high mutation rates (28%); group 3, EEC with low copy number alterations (39%), also called EEC with no specific molecular profile; and finally, group 4 (serous‐like or copy‐number high) (26%), with a low mutation rate but frequent TP53 mutations. These four types show different clinical, pathologic and molecular features.

##### Urothelial bladder carcinoma

Different molecular classifications are currently being explored. One of them, based on RNA genomewide studies, has characterised two main molecular groups of bladder cancer with prognostic and predictive impact.^97^ Group 1 basal subtype includes a subgroup of claudin‐low and is characterised by expression of the basal cytokeratins CK5, CK14 and CD44. Group 2 luminal subtype has mutations of the fibroblast growth factor receptor 3 (FGFR3) gene and urothelial differentiation markers such as CK20, GATA3 and uroplakins. The luminal subtype has a more favourable prognosis than the basal subtype.^97^ Recently, a prospective multicentre validation study with a 12‐gene signature progression score in non‐muscle‐invasive bladder cancer was demonstrated to have independent prognostic power beyond clinical and histopathological risk factors, and could help in stratifying patients to optimise treatment and follow‐up.^98^ Other molecular alterations have also been described, such as FGFR3 mutations in 80%,^99^ PI3KCA mutations in approximately 20% and mutations in the core promoter region of the human telomerase reverse transcriptase (TERT) gene in up to 85% of bladder tumours. Interestingly, FGFR3 and PIK3CA mutations have been demonstrated as biomarkers on liquid biopsy for disease surveillance for bladder cancer.^100^ Different cell cycle genes may be altered in invasive urothelial carcinoma, mainly tumour suppressor genes, including TP53, p16 and Rb.^101^ Finally, combinations of TP53, p21, p27 and pRb proved to be more precise in classifying bladder cancer patients into risk groups.^102^

### Gene expression heterogeneity and environmental cells

#### mRNA expression versus protein expression

Molecular tumour subtypes based on mRNA expression profiles often show discordance with protein expression levels, depending on the RNA stability and the area sampled. It is well known that RNA studies are problematic regarding reproducibility, and that protein expression shows the real functional status of the targets. A classic example is breast cancer, in which mRNA profiles can define at least six different molecular categories. While this can be used as a general classification scheme, only IHC or FISH analysis of proteins in tumour cells within the individual subgroup can provide the exact diagnosis to guide treatment and prognosis. Some reports^67^ describe discord, when comparing mRNA and protein expression levels, of up to 50% of cases in HER2 status, and in more than 15% of cases when distinguishing between luminal A and luminal B.

#### Tissue‐specific protein expression profiles

The relation between tissue context and protein expression has recently been reported in an extensive gene expression study of 17 different tumour types in more than 8000 patients.^103^ The study encompassed massive studies of sequencing, mRNA expression, protein expression and engaged databases from the TCGA and the Human Protein Atlas. The authors also carried out in‐depth bioinformatics and systems biology analysis. Interestingly, more than 2700 genes correlated with prognosis, showing opposite prognostic effects depending on tumour type and location. Within the genes associated with worse prognosis, two groups were described. The first group comprised genes related to cell proliferation, of which more than 190 were identified (of the 314 described related to the cell cycle). This gives an idea of the great redundancy of gene alterations, especially the level of expression that may exist depending on tumour type. The second group comprised genes associated with tissue differentiation and their prognostic correlation. This is a point that has always been described in pathology, distinguishing tumours with a low degree of malignancy, i.e. histologically better differentiated, from those with a high degree of malignancy, i.e. poorly differentiated with a higher degree of cellular atypia. The study also found that more than 50% of the genes associated with prognosis were not related to Hanahan and Weinberg's classic hallmarks.^6^ Therefore, one of the conclusions was the enormous variation in gene expression profiles, both at an inter‐ and intratumour level. They described new sets of genes that, according to their expression, were associated with prognosis in the different tumour types, underlining the clinical importance and the validity of protein expression versus mRNA or genetic alterations. For example, in lung carcinoma, they proposed a prognostic correlation with the expression of the genes ERO1A, S100A16, S100A6, MKi67, SLC2A1, TACC3, ANLN, and CADM1.^103^, ^104^, ^105^

#### Assessment of intratumour heterogeneity based on protein expression

Currently, the most robust readout for ITH assessment comes from IHC. A classic example is ITH of oestrogen receptors, which may be positive in just 1% of cells; in fact, this ITH has been reported to be associated with long‐term risk of fatal breast cancer.^106^ Other examples are the heterogeneous expression of cyclin D1 in mantle cell lymphomas, which otherwise carry a homogeneous CCND1–IGH translocation^107^ and the subtype of HER2+ breast or gastric tumours that can range from 10% to 100% positive cells.^9^, ^108^

Within an entire tumour, protein expression often depends on local factors such as hypoxia, nutrient starvation or oxidative stress, independently of the cell's genetic background. These environmental cues can alter entire signalling pathways downstream of well‐established oncogenic alterations.^109^ Many tumours may have activating mutations in the most upstream elements of certain signalling cascades, such as RAS, EGFR and HER2, but phosphorylation of downstream factors such as MAP‐kinases or mTOR may be inhibited by environmental factors;^110^ therefore, even if specific driver genetic alterations are inhibited, no effect will be observed in cells in which these signalling pathways are inhibited by such factors.^109^, ^111^

#### Evaluation of heterogeneity in immune checkpoints and inflammatory cells

Primary tumours have heterogeneous epithelial cell content, and form an ecosystem with the environmental cells, including stromal cells, endothelial cells, leucocytes, lymphocytes, histiocytes and their secreted factors. In a pathological context, we can quantify and evaluate the expression of the immune checkpoints programmed cell death ligand 1 (PD‐L1) and programmed cell death‐1 (PD‐1) in epithelial and inflammatory parts of the tumour and use this information for clinical correlation studies. Similar to cell signalling pathways, key components of immune checkpoints, such as PD‐L1, can also have heterogeneous expression throughout the tumour.^112^ The PD‐L1 protein is expressed in a wide range of cell types, including lymphocytes, macrophages and dendritic cells, and is stimulated by variable and complex mechanisms. In contrast to other immune checkpoint components, PD‐L1 is rarely expressed in normal tissue but is induced in tumour‐associated tissue, is a tumour biomarker and is also an attractive drug target.^113^, ^114^ As many current treatment schemes are based on immunotherapy, pathological assessment should include immune checkpoint proteins. Furthermore, the heterogeneous and dynamic expression of factors such as PD‐L1 in basal conditions and in response to different treatments presents a challenge to predicting which patients will respond to anti‐PDL1 therapies. The assessment of CD4^+^, CD8^+^ cells, and tumour mutational burden (TMB) can also be helpful in clinical decision‐making. TMB has recently been proposed as a new biomarker to quantify the potential tumoral neoantigenicity, with the aim of predicting patients who will benefit from immunocheckpoint inhibitors. Nevertheless, different technologies and different thresholds have been proposed to define high versus low TMB in different tissue samples.^115^, ^116^, ^117^

Finally, the role of the microenvironment in carcinogenesis is gaining importance, although in small biopsies it is impossible to integrate the role of leucocytes, stromal cells and lymphocytes and the assessment of immune checkpoints.

## Discussion

### The Solution: To Integrate Clinical, Radiological, Molecular and Expression Data Within a Tissue Context

We postulate that cancer formation is a consortium of tumour cell clones, microenvironmental cells and local stressors (such as hypoxia) that alter protein expression and enhance tumour heterogeneity. Therefore, we believe that cancer must be considered within the context of tissue type. The correct diagnosis of a tumour should assimilate the clinical, radiological, histopathological, molecular and proteomic data.

As intratumour protein and molecules are highly heterogeneous and diagnostic biopsies are small, we envision that correlation with radiological imaging and nuclear medicine will be crucial to select areas that are more representative of the whole tumour.

Determination of the exact histological type is crucial for an exact diagnosis; for example, in lung carcinoma there is a huge difference in prognosis and treatment between diagnoses of small‐cell carcinoma, adenocarcinoma or large‐cell carcinoma. Similarly, in ovarian carcinoma, prognosis and survival varies widely between types: high‐ or low‐grade serous carcinoma, endometrioid carcinoma, clear‐cell carcinoma or mucinous adenocarcinoma. Most tumours can be diagnosed according to histological criteria as high‐ or low‐grade. The distinction is based on the degree of cell differentiation, the intensity of cellular atypia and cell proliferation. A proliferation index, such as Ki67, can be used to quantify the percentage of replicative cells within a tumour. This simple protein can be detected regardless of which genetic alterations are inducing the proliferation. The above‐mentioned classical factors have all been validated by RNA and molecular data.^103^

#### Identification of master genes and funnel factors

As described above, cellular stress can alter gene expression in tumour cells at several levels modifying biochemical signalling pathways and feedback loops,^111^ and protein expression can be heterogenous. While these biochemical changes are restricted to certain areas within the tumour, funnel factors such as p4E‐BP1 and peIF4E are usually expressed evenly throughout the tumour, regardless of the upstream oncogenic alterations and local environmental changes. The search for cellular targets expressed in most areas of tumours is becoming crucial to overcome tumour heterogeneity.

Because of the huge amount of data and complex positive and negative feedback regulating the final signal and the biological effect, systems biology is becoming essential for the understanding and identification of the real nodes or factors which are drivers in specific tumours in individual patients. The proto‐oncogene myc in Burkitt lymphoma and the canonical nuclear factor kappa B heterodimer in diffuse large B cell lymphoma are classic examples of so‐called master genes.^118^, ^119^, ^120^

#### Advances in digital pathology

Advances in digital pathology (whole slide imaging) now allow interpretation of digital slides on computer stations and integration of molecular features into the microscopic context. The differential expression of genes in different areas of the tumour can be interpreted precisely, and haematoxylin and eosin‐stained microscopic sections can be merged with multiple immunohistochemical stains for different proteins. Digital pathology also allows tumour annotation for microdissection for different tumour components, with appropriate cellularity assessment of tumour elements and the non‐neoplastic microenvironment. Digital pathology is essential to establish computerised algorithms to correlate molecular alterations with tumour phenotype.

#### Tissue‐specific multiplex technology

Given the numerous alterations in genes, protein expression and mRNA, it is clear that we will have to implement multiplex technologies that will allow us to study multiple factors simultaneously on the same histological sections. With this approach we can minimise the amount of tissue needed for molecular and protein studies and for quantification of the expression of different factors and their inter‐relationship. Different platforms are emerging, with different quantitative multispectral fluorescence approaches, or with mass spectrometry *in situ*, to allow the study and quantification of several dozen markers. Also, new next‐generation sequencing technologies are being implemented in routine testing combining DNA and RNA analysis from the same slide reducing the amount of tissue and also the ITH.^121^, ^122^

#### An interpretative and holistic data assessment with ‘deep learning’ (artificial intelligence with automatic learning)

**Figure 2:**
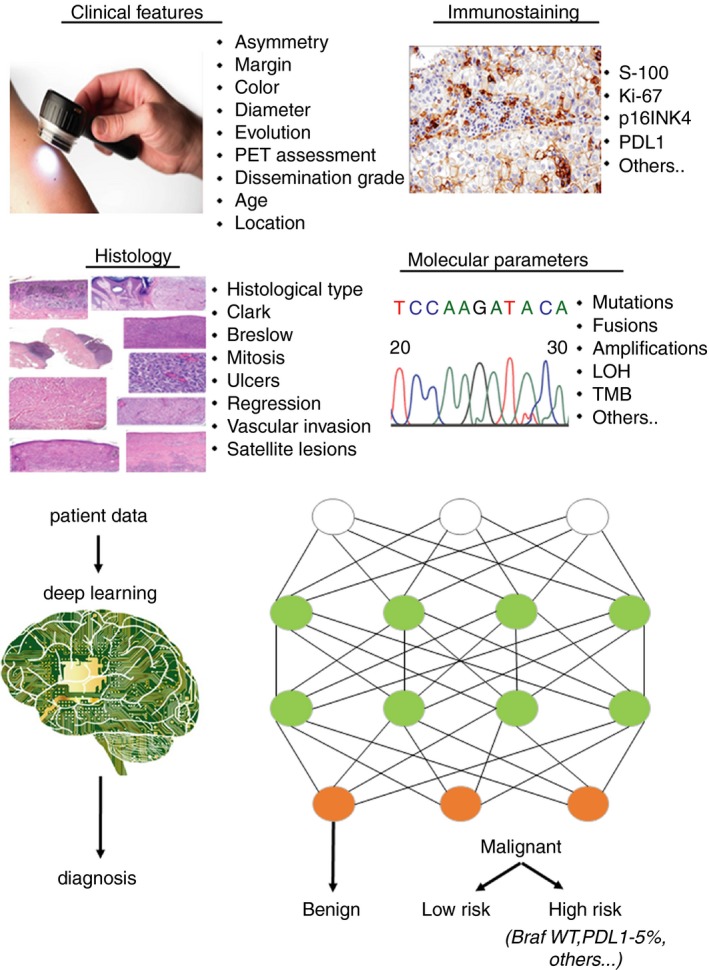
Schematic representation of the potential use of artificial intelligence (AI) in the diagnosis of melanoma. All information (clinical features, histology, molecular alterations, other parameters) are analysed through AI establishing different algorithms resulting in a more precise diagnosis.

The main objective for the use of deep learning in pathology is to improve existing algorithms for biomarker quantification and to develop smart and robust algorithms. It can be based on three main areas or objectives: (i) tumour type classification, (ii) tumour prognosis (prediction system, learning from experience) and (iii) personalised treatment allocation system (also a reinforcement learning system). In fact, deep learning is already being applied in various tumour types, including breast, lung, melanoma and glioblastoma.^123^, ^124^, ^125^, ^126^ The integration of such information and algorithms into everyday clinical practice can help to minimise subjective evaluations and improve the precise diagnosis for each tumour and patient (see Figure 2).

Systems biology encompasses tools that hold great promise for deciphering the vulnerabilities of the tumour ecosystem as a whole. Studies are based on the premise that multiple oncogenic events converge on a number of cellular networks that may contain essential or synthetically lethal clinically targetable hubs or factors,^127^, ^128^ such as the eIF4F complex. Nonetheless, many contemporary systems biology approaches do not consider intratumour heterogeneity. This is relevant because key functional nodes within the metabolic, signal transduction and gene expression networks responsible for supporting the tumour phenotype are critically dependent on the heterogeneity of the tumour. These data can also help to classify tumours according to histopathological, biochemical and genomic features and thus help to tailor the diagnosis and clinical management to a patient's biological tumour profile. Accordingly, multidimensional molecular and gene expression data, which are associated with the response to antitumour treatments and clinical progress, are thought to facilitate the selection of patients who are more likely to respond to targeted or ‘precise’ therapies.^129^, ^130^, ^131^ The availability of systemwide data in a variety of cancers is facilitating the development of approaches that go beyond the classic, reductionist paradigms that associate single genes with cellular phenotypes and functions. Systems biology approaches consider the interplay between multiple molecular factors that underpin phenotype development. Accordingly, rather than a genetic disease, cancer is now perceived as a disease of ‘networks’,^131^ and to fully grasp the complexity of the tumour ecosystem the networks driving cancer need to be mapped and their dynamics and evolution over time deciphered. Emerging data show that these cancer networks are constantly rewired in part by clonal interactions, changing microenvironments and the acquisition of novel molecular alterations that are largely induced by anticancer treatments.^127^, ^132^ Therefore, minor subpopulations that are not readily detectable in bulk tumours or that can adapt to hostile environments may emerge following treatments that specifically target cancer‐driving mutations present in the predominant tumour subpopulations. Thus, in the coming years, systems biology implementation may be crucial for (i) detailed, clinically oriented subclassification of cancer types, (ii) mapping of oncogenic networks and identification of the critical nodes to overcome the effects of ITH and (iii) informing future preclinical research and design of Phase I clinical trials by anticipating therapy response *in silico* and predicting the best targets for each patient and tumour (personalised cancer therapy or precision medicine).

#### Liquid biopsies

As biopsies are associated with a degree of invasiveness and cannot always be repeated, new approaches have been proposed, such as the analysis of circulating tumour cells (CTCs), plasma‐derived cell‐free tumour DNA (cfDNA) or RNA and, more recently, DNA from circulating tumour exosomes in blood.^133^, ^134^, ^135^ This strategy enables a global assessment of the constellation of somatic genetic alterations in a tumour irrespective of its anatomical location, and is becoming a good alternative for the study of a tumour mutational landscape in situations where biopsies or surgical samples are difficult to obtain. Hence, liquid biopsy appears to be a very relevant tool for the identification of potential actionable genomic alterations and therapeutic targets, monitoring treatment response, predicting disease progression before clinical and radiological confirmation and identifying mechanisms of resistance in the particular context of patients with early‐stage and metastatic disease.^136^, ^137^ Furthermore, liquid biopsy provides additional cues on ITH, as reported in a recent pilot study in patients with early‐stage NSCLC.^138^

## Conclusion

**Figure 3:**
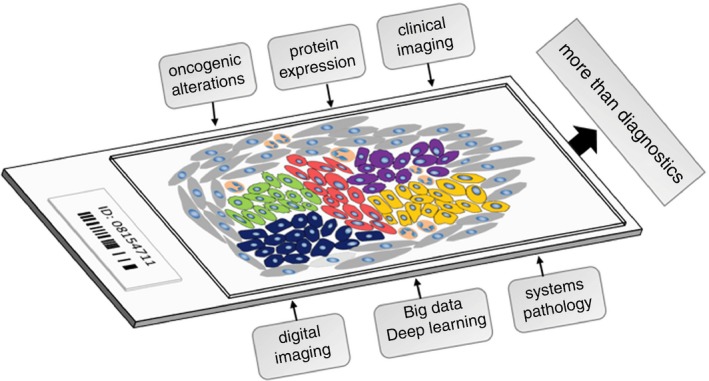
Schematic drawing of a tumour section for histopathological analysis. Heterogeneity within a tumour is depicted by different colours of the tumoral cells. Stromal cells and infiltrating cells of the immune system are coloured in grey and orange, respectively. Variables considered for the evaluation of the tumour sample are indicated in the boxes above the slide. New methodologies, proposed to improve the diagnosis, are shown in the boxes below.

Tumours show such a degree of morphological, molecular and proteomic heterogeneity, and a close relationship with microenvironmental factors, that for the accurate pathological evaluation of tumours an integrated approach seems essential: all genomic and proteomic data should be evaluated taking into account the heterogeneity and tissue type (Figure 3). We propose the integration of all such factors, along with the classical pathological factors, within the context of the tissue; we call this approach *tissunomics*.

## Conflicts of interest

All authors declare no conflicts of interest.
